# Low-temperature synthesis of LiFePO_4 _nanocrystals by solvothermal route

**DOI:** 10.1186/1556-276X-7-3

**Published:** 2012-01-05

**Authors:** Jinsub Lim, Sung-Won Kang, Jieh Moon, Sungjin Kim, Hyosun Park, Joseph Paul Baboo, Jaekook Kim

**Affiliations:** 1 Department of Materials Science and Engineering, Chonnam National University, 300 Yongbongdong, Bukgu, Gwangju 500-757, South Korea

**Keywords:** low-temperature synthesis, nanocrystals, LiFePO_4_, cathode, lithium batteries

## Abstract

LiFePO_4 _nanocrystals were synthesized at a very low temperature of 170°C using carbon nanoparticles by a solvothermal process in a polyol medium, namely diethylene glycol without any heat treatment as a post procedure. The powder X-ray diffraction pattern of the LiFePO_4 _was indexed well to a pure orthorhombic system of olivine structure (space group: Pnma) with no undesirable impurities. The LiFePO_4 _nanocrystals synthesized at low temperature exhibited mono-dispersed and carbon-mixed plate-type LiFePO_4 _nanoparticles with average length, width, and thickness of approximately 100 to 300 nm, 100 to 200 nm, and 50 nm, respectively. It also appeared to reveal considerably enhanced electrochemical properties when compared to those of pristine LiFePO_4_. These observed results clearly indicate the effect of carbon in improving the reactivity and synthesis of LiFePO_4 _nanoparticles at a significantly lower temperature.

## Introduction

The orthorhombic olivine structure of LiFePO_4 _is intensively investigated as the most attractive cathode to replace commercialized LiCoO_2 _in rechargeable lithium-ion batteries because it is not only inexpensive, nontoxic, and environmentally benign, but has also a relatively high theoretical capacity of 170 mAh g^-1 ^and a suitably flat voltage region of 3.45 V within the electrolyte window [1]. However, despite these advantages, its unimpressive rate performance due to intrinsic problems of low ionic and electronic conductivities still remains as a major obstacle for commercial applications. Progressive efforts to circumvent this obstacle by carbon coating on particle surface [2], developing composites via mixing conductive materials [3], aliovalent cation substitution [4], particle size minimization [5,6], and customizing particle morphologies [7-11] have been undertaken.

Among these approaches, with respect to Li-ion batteries, nano-sized electrodes have been intensively investigated for high-power density applications as the advantage of using such electrodes remains twofold [12,13]. Firstly, nanomaterials provide a favorable structural framework that ensures shorter diffusion paths for the Li-ions to traverse from the core of the particles to the surface through the lattice, thereby yielding excellent electrochemical properties. Secondly, the large surface area of nanomaterials ensures enhanced electrode/electrolyte interfacial contact, thus leading to higher charge/discharge rates and good capacity retentions [14,15]. In addition, it is also evident that customizing particle morphologies is becoming highly important since these factors are well known to significantly influence material properties and electrochemical performances.

Ultimately, designing nanomaterials is a critical issue for the industrial applications because device performance is hugely dependent on material properties. In addition, the efficacy of technologies for nanomaterials largely depends on preparation and processing methods. To date, various synthesis techniques have been adopted for synthesizing nano-sized LiFePO_4 _and controlling the morphology of LiFePO_4 _nanoparticles by soft chemistry processes such as co-precipitation [16], sol-gel [17], hydrothermal [7,8,18], polyol [11,15], and solvothermal methods [9,10,19,20]. Our group has introduced inexpensive and simple processes of conventional polyol method, microwave-assisted polyol process and solvothermal method using the polyol media, which are new methods of synthesizing LiFePO_4 _with well-defined nanoparticles and high crystallinity, without the need for any further heating as a post-treatment [15,19,21,22]. In these processes, the polyol media play an important role not only as solvents, but also as a reducing environmental agent and stabilizer, thereby limiting particle growth and preventing agglomeration [15]. Among these processes, the solvothermal process is especially advantageous for synthesizing LiFePO_4_, which can obtain nanoparticles with high crystallinity at a relatively low temperature because of high pressure at a moderate temperature, compared with conventional and microwave-assisted polyol process. Also, we have reported that nano-sized LiMPO_4_(M = Fe, Mn, Co) has been synthesized at a reaction temperature of 200°C by solvothermal method in which the LiFePO_4 _sample has showed good electrochemical properties [19,20,23]. When we have used various polyol media of ethylene glycol [EG], diethylene glycol [DEG], triethylene glycol [TEG], and tetraethylene glycol [TTEG] for synthesizing LiFePO_4 _nanoparticles at a temperature of 200°C, it was confirmed that reactivity might be affected because a pressure in the bomb during the solvothermal reaction is different according to the boiling point of the polyol media [24].

In this work, we report synthesizing nanostructured LiFePO_4 _with an olivine structure and high crystallinity by solvothermal method employing carbon nanoparticles at a very low temperature of 170°C. The physical properties and electrochemical performances of the obtained LiFePO_4 _samples were investigated.

## Method

### Synthesis

#### LiFePO_4 _without carbon

LiFePO_4 _was synthesized by solvothermal process as follows: (C_2_H_3_O_2_)_2_Fe (99.995%; Sigma-Aldrich, Yongin-city, Kyunggi-do, South Korea), H_3_PO_4 _(above 85%; Daejung Chemicals & Metals Co., Ltd, Shiheung-city, Gyeonggi-Do, South Korea), and CH_3_COOLi·2H_2_O (GR, Junsei Chemical Co., Ltd, Tokyo, Japan) were added to (HOCH_2_CH_2_)_2_O (99%; Daejung Chemicals & Metals Co., Ltd) in the molar ratio 1:1:1 (Li:Fe:P) and stirred for about 30 min at room temperature. The mixed solution was transferred into a Teflon-lined bomb with a volume of 40 ml. The bomb was sealed and heated at mild temperatures for 16 h in a high-temperature conventional oven.

#### Carbon-mixed LiFePO_4_

CH_3_COOLi·2H_2_O (GR, Junsei Chemical Co., Ltd), (C_2_H_3_O_2_)_2_Fe (99.995%, Sigma-Aldrich), H_3_PO_4 _(above 85%; Daejung Chemicals & Metals Co., Ltd), and diethylene glycol (99%; Daejung Chemicals) were used as the starting materials, and Ketjen Black was used as a carbon source. Initially, 10 wt% Ketjen Black was added in a 25-ml DEG solvent and treated with ultrasonic for 1 h. Subsequently, Fe, Li, and P precursors were added to the solution and stirred for 30 min at room temperature. The mixed solution was transferred into a Teflon-lined bomb with a volume of 40 ml. The bomb was sealed and heated at 170°C for 16 h in a high-temperature conventional oven.

After the solvothermal reaction, the resulting solutions containing grayish precipitates were washed with acetone and methanol several times in order to ensure removal of the organic compounds, respectively. The obtained precipitates were separated by filtering using ceramic membrane funnels and dried at 120°C for 12 h in a vacuum oven.

### Structural and physical characterization

Powder X-ray diffraction [XRD] data of the obtained samples were recorded using a Shimadzu X-ray diffractometer with Ni-filtered Cu Kα radiation (*λ *= 1.5406 Å) operating at 40 kV and 30 mA within the scanning angle of 2*θ*, range 15° to 60° in steps of 0.02°. The particle morphologies and sizes were determined by field emission-scanning electron microscopy [FE-SEM].

### Electrochemical characterization

The electrochemical properties of the LiFePO_4 _samples were evaluated with lithium metal as the reference and counter electrode. For the electrochemical measurements, the active material was mixed with conductive carbon (Ketjen Black), and Teflonized Acethylene Black was used as a binder. The mixture was pressed onto a stainless steel mesh and vacuum dried at 120°C for 12 h, thus forming the cathode. A 2032 coin-type cell consisting of the cathode and lithium metal anode separated by a polymer membrane together with glass fiber was fabricated in an Ar-filled glove box and aged for 12 h before the electrochemical measurements. The electrolyte employed was a 1:1 mixture of ethylene carbonate and dimethyl carbonate containing 1 M LiPF_6_.

## Results and discussion

**Figure 1:**
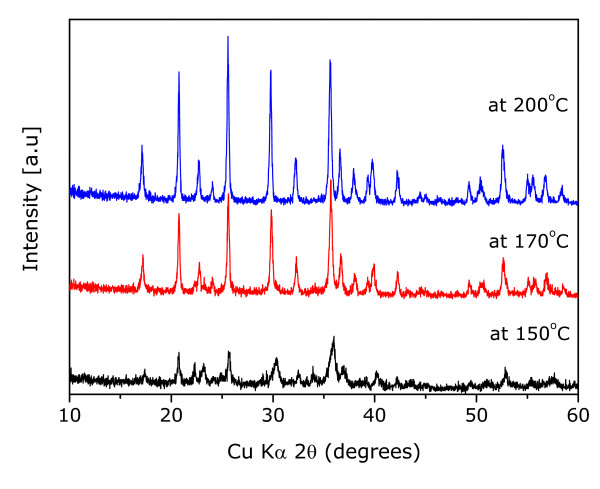
**X-ray diffraction patterns of LiFePO_4 _samples prepared at various temperatures by solvothermal technique**.

Figure 1 shows X-ray diffraction patterns of the obtained LiFePO_4 _samples synthesized at various temperatures of 150°C, 170°C, and 200°C using DEG through the solvothermal reaction. In the case of the sample synthesized at 200°C, all of the peaks are indexed on the basis of an orthorhombic olivine-type structure (space group: Pnma), and no second phase such as Li_3_PO_4 _or transition metal compounds was observed. Although the XRD pattern of the sample synthesized at 170°C showed mainly an olivine phase, it was revealed that second phases existed, such as Li_3_PO_4 _and Fe_3_(PO_4_)_2_, because of deficiency of heat energy. Notably, the XRD pattern of the sample that reacted at 150°C appears to have a much stronger intensity of impurity peaks such as Li_3_PO_4 _and Fe_3_(PO_4_)_2 _than that at 170°C, indicating that the reaction for the olivine nanoparticles did not occur sufficiently below 170°C.

**Figure 2:**
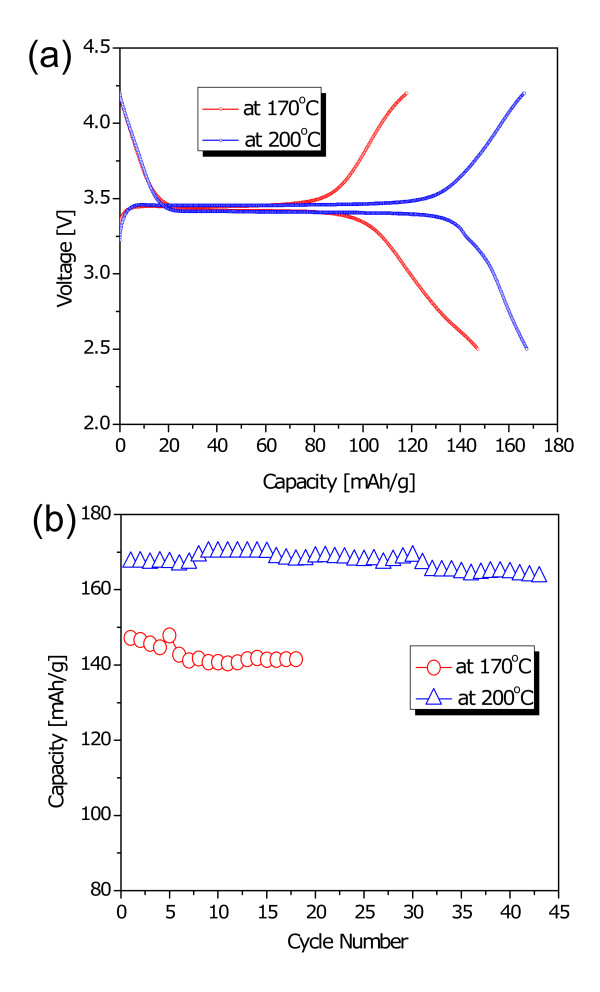
**The electrochemical performances of the LiFePO_4 _samples synthesized at 170°C; and 200°C**. (**a**) Initial charge/discharge curves at a current density of 0.1 mA cm^-2 ^in the voltage range of 2.5 to approximately 4.2 V and (**b**) the charge/discharge capacity retention rates.

Due to these reasons, electrochemical tests were conducted for only samples prepared at 170°C and 200°C. Figures 2a and 2b revealed the voltage profiles of LiFePO_4 _electrode materials in the first cycle and cyclabilities, respectively, under a current density of 0.1 mA cm^-2 ^in the voltage range of 2.5 to 4.2 V. The first discharge capacities of the LiFePO_4 _synthesized at 170°C and 200°C were registered to be 149 and 168 mAh g^-1^, respectively. Interestingly, the initial discharge capacity of the sample that reacted at 200°C was close to 99% of its theoretical capacity with almost no capacity fade during the extended cycles. However, the sample at 170°C revealed a much lower discharge capacity than the other sample because of the impurities of Li_3_PO_4 _and Fe_3_(PO_4_)_2_, as evidenced from XRD patterns in Figure 1.

In our earlier paper, we showed that a pressure in the bomb during the solvothermal reaction at the same temperature might differ according to the polyol media of EG, DEG, TEG, and TTEG [24]. Notably, among these polyol media, DEG was the most suitable for synthesizing the LiFePO_4 _nanoparticles at the same reaction temperature of 200°C due to the appropriate boiling point of 240°C. In terms of economics, lowering reaction temperature during synthesizing is one of the critical points in commercialization. Therefore, we have tried to lower the reaction temperature during solvothermal process using carbon and simultaneously obtain the olivine nanoparticles with high crystallinity.

**Figure 3:**
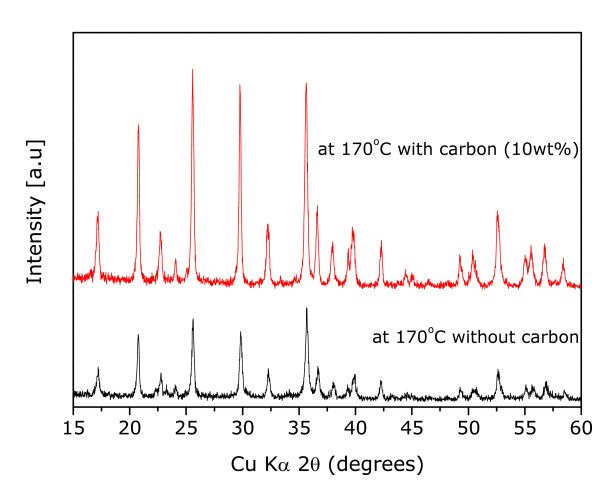
**X-ray diffraction patterns of pristine and carbon-mixed LiFePO_4 _samples synthesized at 170°C**.

**Figure 4:**
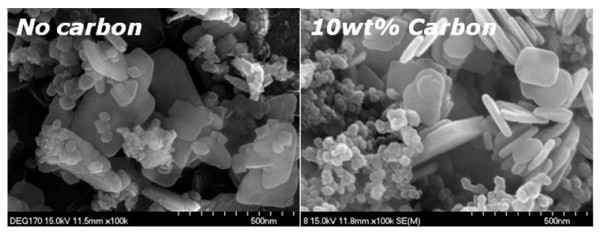
**The field-emission SEM images of pristine and carbon-mixed LiFePO_4 _samples synthesized at 170°C**.

**Figure 5:**
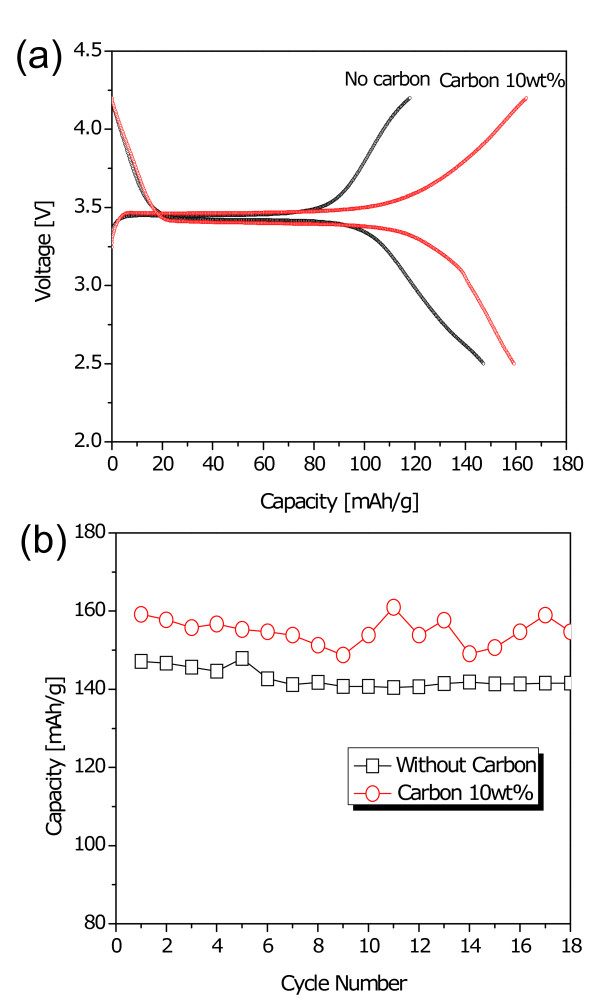
**The electrochemical performances of pristine and carbon-mixed LiFePO_4 _samples**. (**a**) Initial charge/discharge curves at a current density of 0.1 mA cm^-2 ^in the voltage range of 2.5 to approximately 4.2 V and (**b**) cyclability.

Carbon can suppress particle growth during reaction resulting in a decrease in aggregated state [25] and an increase in the reactivity. In Figure 1, LiFePO_4 _sample synthesized at 170°C showed mainly an olivine phase, even if there was a little revelation that second phases existed, such as Li_3_PO_4 _and Fe_3_(PO_4_)_2_. We have improved properties of the sample that reacted at 170°C by modification using a conduction material of Ketjen Black. Figure 3 shows X-ray diffraction pattern of the reacted sample with an amount of 10 wt% Ketjen Black prepared at a significantly low temperature of 170°C. Even though the sample without carbon revealed little impurity peaks in XRD pattern, all impurities disappeared, and peak intensities increased after reaction using carbon. In order to observe the particle size and morphology of the sample prepared by the solvothermal method, field-emission SEM was used. In the case of the sample with 10 wt% carbon, it showed mono-dispersed and carbon-mixed plate-type LiFePO_4 _nanoparticles with average length, width, and thickness of approximately 100 to 300 nm, 100 to 200 nm, and 50 nm, respectively, as shown in Figure 4. In order to certify better rate capability caused by particle size minimization and carbon effect in the LiFePO_4 _nanoparticles, we performed galvanostatic test using the carbon-mixed LiFePO_4 _and pristine LiFePO_4 _for comparison purposes. Figures 5a and 5b reveal the voltage profiles of a modified LiFePO_4 _nanoparticle in the first cycle and cyclabilities, respectively, under a current density of 0.1 mA cm^-2 ^in the voltage range of 2.5 to 4.2 V. The first discharge capacity of the synthesized LiFePO_4 _with 10 wt% carbon at 170°C was registered to be 160 mAh g^-1 ^which was close to 94% of its theoretical capacity. These results clearly indicated the effect of the addition of carbon in improving the reactivity and synthesis of LiFePO_4 _nanoparticles at a significantly lower temperature.

## Conclusions

LiFePO_4 _nanocrystals with high crystallinity were synthesized at a very low temperature of 170°C using carbon nanoparticles by a solvothermal process in a polyol medium, namely DEG without any heat treatment as a post procedure. In XRD pattern, all the peaks are indexed on the basis of an orthorhombic olivine-type structure (space group: Pnma), and no second phase such as Li_3_PO_4 _or transition metal compounds was observed though the sample without carbon revealed little impurity peaks. The LiFePO_4 _nanocrystals synthesized at low temperature exhibited mono-dispersed and carbon-mixed plate-type LiFePO_4 _nanoparticles with average length, width, and thickness of approximately 100 to 300 nm, 100 to 200 nm, and 50 nm, respectively. In order to certify better rate capability caused by particle size minimization and carbon effect in the LiFePO_4 _nanoparticles, galvanostatic test was performed using the carbon-mixed LiFePO_4 _and pristine LiFePO_4 _without carbon for comparison purposes. Further, the carbon-mixed LiFePO_4 _sample appeared to reveal considerably enhanced electrochemical properties when compared to those of pristine LiFePO_4_. Thus, the observed results clearly indicate the effect of carbon in improving the reactivity and significantly low-temperature synthesis of LiFePO_4 _nanoparticles.

## Competing interests

The authors declare that they have no competing interests.

## Authors' contributions

JK directed the research. JL wrote this manuscript. SWK and JM analyzed the data. SK and HP prepared the LiFePO_4 _materials and carried out the experimental measurements. JPB contributed to the technical discussions.
